# Clinical and Microbiological Aspects of Colistin Resistance in Klebsiella pneumoniae Isolates From Tertiary-Care Hospital

**DOI:** 10.7759/cureus.110565

**Published:** 2026-06-09

**Authors:** Shivraj Jadhav, Geeta Karande, Satish Patil

**Affiliations:** 1 Department of Microbiology, Krishna Institute of Medical Sciences, Krishna Vishwa Vidyapeeth (Deemed to be University), Karad, IND

**Keywords:** antimicrobial susceptibility testing, colistin resistance, klebsiella pneumoniae, multidrug-resistant bacteria, phenotypic pattern

## Abstract

*Klebsiella pneumoniae *is a major opportunistic pathogen responsible for a wide range of hospital-acquired infections such as pneumonia, urinary tract infections, bloodstream infections, and septicemia. The increasing occurrence of multidrug-resistant (MDR) and carbapenem-resistant strains has led to the reintroduction of colistin as an antibiotic of last resort used to treat severe infections caused by Gram-negative bacteria. However, the emergence of colistin resistance has emerged as a significant global public health concern, particularly in tertiary-care hospital settings, as it restricts available treatment options and complicates infection control measures. This review emphasizes the phenotypic patterns, underlying mechanisms, risk factors, and clinical importance regarding colistin resistance in *K. pneumoniae* isolates. Phenotypically resistant strains generally show minimum inhibitory concentration (MIC) values of ≥4 µg/mL. Resistance mechanisms include lipopolysaccharide modification, plasmid-mediated *mcr *genes, biofilm formation, and efflux pump activity. Risk factors such as extended hospital stays, admission to intensive care units, previous antibiotic use, and the utilization of invasive medical devices contribute to the development and dissemination of resistant strains. Ongoing surveillance, precise antimicrobial sensitivity testing using the broth microdilution method, and effective infection control practices are crucial to minimizing the burden of colistin-resistant infections in tertiary-care hospitals.

## Introduction and background

*Klebsiella pneumoniae* is a rod-shaped, encapsulated, non-motile, Gram-negative bacterium classified under the Enterobacteriaceae family and is widely distributed in environmental sources such as soil, water, and plants, as well as in the gastrointestinal tracts of humans and animals, where it exists as part of the normal intestinal flora [1]. The bacterium is recognized for its remarkable adaptability and ability to survive under diverse environmental and host-associated conditions, which contribute significantly to its persistence in both community and healthcare settings. Due to its ability to colonize mucosal surfaces and survive on contaminated medical equipment and hospital environments, *K. pneumoniae* has become an increasingly important healthcare-associated pathogen worldwide [1].

Although it is generally considered a commensal organism in healthy individuals, *K. pneumoniae* has emerged as an important opportunistic pathogen responsible for a broad spectrum of infections, particularly among immunocompromised individuals, hospitalized patients, and critically ill individuals admitted to intensive care units (ICUs). The organism is associated with a variety of infections, including bloodstream infections, meningitis, urinary tract infections (UTIs), pneumonia, septicemia, wound infections, liver abscesses, and healthcare-associated infections [2]. Patients with prolonged hospitalization, diabetes mellitus, chronic diseases, malignancies, weakened immune systems, or invasive medical devices such as urinary catheters and mechanical ventilators are particularly vulnerable to infections caused by this organism [2]. In recent decades,* K. pneumoniae* has increasingly been implicated in hospital-acquired infections, contributing substantially to patient morbidity, mortality, prolonged hospitalization, and increased healthcare expenditure.

The bacterium was first recognized as a cause of pneumonia in the late 19th century by Carl Friedländer, who identified the pathogen in patients suffering from severe pulmonary infections, which led to its earlier designation as Friedländer’s bacillus [3]. Since its discovery,* K. pneumoniae *has become one of the most clinically important bacterial pathogens in both developed and developing nations. During the 20th century, the widespread use of antibiotics significantly reduced deaths associated with bacterial infections and transformed modern medicine by improving patient outcomes. However, the excessive and inappropriate use of antimicrobial agents over time contributed to the gradual emergence of multidrug-resistant (MDR) strains of* K. pneumoniae*, which has become a major clinical concern worldwide. The increasing burden of antimicrobial resistance (AMR) among Gram-negative bacteria poses a significant challenge to public health because it limits therapeutic options and complicates infection management in healthcare settings [3].

Among the antimicrobial agents used against MDR Gram-negative pathogens, colistin, a member of the polymyxin group of antibiotics introduced into medical practice in the 1950s, gained considerable attention because of its strong bactericidal activity against resistant organisms. Colistin acts primarily by disrupting the integrity of the bacterial outer membrane through interactions with lipopolysaccharides (LPSs), ultimately resulting in bacterial cell death. Initially, the antibiotic was highly effective in treating severe infections caused by Gram-negative bacteria. However, its routine clinical use declined substantially during the 1980s due to concerns regarding nephrotoxicity and neurotoxicity, which limited its therapeutic acceptance for several decades [4].

Despite concerns regarding toxicity, the global emergence of carbapenem-resistant and MDR Gram-negative pathogens in the early 2000s led to the reintroduction of colistin as a last-resort therapeutic option for severe infections caused by organisms such as* K. pneumoniae*. In many tertiary-care hospitals and intensive care settings, colistin regained importance because alternative antimicrobial options became increasingly limited. Nevertheless, the increasing use of colistin has subsequently contributed to the emergence of colistin-resistant *K. pneumoniae* strains, creating an additional challenge in clinical treatment and infection control. The development of resistance to colistin is especially concerning because it compromises one of the final available therapeutic options for managing infections caused by MDR bacteria [4].

The detection and characterization of colistin resistance in clinical isolates are essential for guiding appropriate antimicrobial therapy and implementing effective infection prevention and control strategies. AMR has emerged as a major global public health concern because resistant organisms are associated with higher morbidity, mortality, treatment failure, and healthcare costs. In tertiary-care hospitals, where critically ill patients frequently require prolonged hospitalization and broad-spectrum antimicrobial therapy, the emergence and dissemination of resistant strains are particularly problematic. Therefore, understanding the clinical and microbiological characteristics of colistin resistance in *K. pneumoniae* is necessary to improve diagnostic approaches, optimize antimicrobial treatment strategies, and strengthen antimicrobial stewardship programs (ASPs).

Furthermore, *K. pneumoniae* possesses virulence factors that include a polysaccharide capsule, biofilm formation, siderophore-mediated iron acquisition, and adhesins that enhance immune evasion, colonization, and persistence in the host that contribute to its pathogenicity and survival within the host. One of the most important virulence determinants is its well-developed polysaccharide capsule, which protects the organism from phagocytosis and host immune responses, thereby enhancing bacterial survival and virulence. Additional virulence-associated mechanisms, including biofilm formation, iron acquisition systems, and adhesins, further facilitate colonization, persistence, and infection in susceptible individuals [5]. These pathogenic characteristics, together with increasing AMR, have made *K. pneumoniae* a major challenge in modern clinical practice and an important focus of ongoing microbiological and infectious disease research [5].

## Review

A literature search was conducted using electronic databases such as PubMed (Bethesda, Maryland), Google Scholar (Mountain View, California), Scopus (Amsterdam, the Netherlands), and Web of Science (London, United Kingdom) to identify studies on colistin resistance in *K. pneumoniae*. Keywords including "*Klebsiella pneumoniae*," "colistin resistance," "mcr genes," "antimicrobial resistance," and "risk factors" were used with Boolean operators (AND, OR) to refine the search. Only relevant English-language articles were included in this review.

Global epidemiology of colistin resistance in* K. pneumoniae*

The global increase in colistin-resistant *K. pneumoniae* has emerged as a significant public health concern and continues to pose a growing challenge in both developed and developing nations, especially within ICUs, tertiary-care hospitals, and specialized referral healthcare centers. The rise and dissemination of colistin resistance have primarily been attributed to the extensive, and in certain settings inappropriate, use of broad-spectrum antimicrobial agents, together with the increasing burden of MDR and carbapenem-resistant Gram-negative bacterial infections [6, 7, 8]. Because colistin is widely regarded as one of the last available therapeutic agents for severe infections caused by carbapenem-resistant organisms, the emergence of resistance carries major clinical consequences by further limiting treatment choices for critically ill patients [6, 7].

Global surveillance data obtained from Asia, Europe, North America, and the Middle East demonstrate substantial regional variation in the prevalence of colistin-resistant *K. pneumoniae*. Such differences in resistance patterns are influenced by several contributing factors, including local antimicrobial prescribing behavior, the effectiveness of ASPs, infection prevention strategies, healthcare infrastructure, and region-specific epidemiological trends [6, 7]. Regions with inadequate stewardship measures and excessive empirical antibiotic administration have generally reported higher resistance rates, highlighting the urgent necessity for coordinated surveillance programs and strengthened infection control interventions [7].

Multiple epidemiological investigations have reported a considerably greater prevalence of colistin-resistant *K. pneumoniae* in Asian countries, particularly in India, China, and several Southeast Asian regions, compared with many Western countries. In India, the emergence of colistin-resistant isolates has increasingly been documented in tertiary care institutions, especially among ICU patients and individuals experiencing prolonged hospitalization or repeated exposure to broad-spectrum antibiotics [1, 2, 8]. The burden of resistance in these healthcare settings is particularly concerning because hospitals frequently manage a high incidence of carbapenem-resistant Enterobacteriaceae (CREs), resulting in greater dependence on colistin as a salvage treatment option [1, 8]. As a consequence, increased reliance on colistin has intensified selective pressure, facilitating the emergence and spread of resistant bacterial strains within hospital environments.

The epidemiological burden of colistin resistance is especially alarming in intensive care settings, where critically ill individuals are more susceptible to resistant infections due to invasive interventions, mechanical ventilation, indwelling urinary catheters, central venous catheterization, immunosuppressive conditions, and prolonged antimicrobial exposure [4, 9]. Outbreaks involving colistin-resistant*K. pneumoniae* have increasingly been observed in tertiary-care referral hospitals and are associated with considerable morbidity and mortality among hospitalized patients [4]. The transmission of resistant organisms within ICUs may occur through direct patient contact, contaminated environmental surfaces, inadequate infection prevention measures, and colonization of healthcare workers or medical equipment. Such outbreaks present substantial difficulties for hospital infection control programs because resistant strains can rapidly disseminate in high-risk healthcare environments [9].

Although surveillance studies from Europe and North America generally report comparatively lower rates of colistin resistance, increasing trends have also been observed, particularly in healthcare institutions involved in the management of MDR infections [6, 7]. The emergence of plasmid-mediated mobilized colistin resistance (*mcr*) genes has further complicated the epidemiological scenario because these resistance determinants possess the ability to spread rapidly through horizontal gene transfer among bacterial populations. This process facilitates dissemination not only among* K. pneumoniae* isolates but also across other clinically important Gram-negative pathogens, thereby increasing the worldwide burden of AMR [8].

From a clinical and healthcare perspective, the growing prevalence of colistin-resistant* K. pneumoniae *significantly reduces the availability of effective treatment options and is associated with adverse clinical outcomes, including prolonged hospitalization, increased treatment failure, elevated healthcare costs, and higher mortality rates [9, 10]. Several studies have indicated that bloodstream infections and ventilator-associated pneumonia caused by resistant strains are especially challenging to treat because of limited antimicrobial choices and delayed microbiological response to therapy [10]. In addition, the financial burden associated with prolonged ICU admissions, advanced laboratory investigations, and combination antimicrobial regimens places considerable strain on healthcare systems, particularly in low and middle-income countries [9]. Considering the increasing global burden of colistin resistance, there is an urgent need to strengthen antimicrobial stewardship initiatives, reinforce infection prevention practices, encourage rational antibiotic prescribing, and maintain continuous epidemiological monitoring to control the spread of resistant *K. pneumoniae* strains [6, 7]. Furthermore, early detection of resistant isolates, combined with comprehensive molecular surveillance, may contribute significantly to outbreak prevention and assist in guiding targeted therapeutic approaches, especially in tertiary-care healthcare facilities where vulnerable patient populations remain at elevated risk of infection [8].

Importance of colistin resistance

Colistin is considered one of the final therapeutic options for the management of severe infections caused by MDR Gram-negative bacteria, particularly *K. pneumoniae*. The increasing emergence of colistin resistance has become an important global healthcare issue because it markedly reduces the efficacy of one of the few remaining antimicrobial agents available for the treatment of life-threatening infections associated with MDR and carbapenem-resistant pathogens [10]. As colistin is commonly reserved for critically ill patients with restricted therapeutic alternatives, the development of resistance to this antibiotic poses serious consequences for both clinical treatment and public health systems globally.

The clinical relevance of colistin resistance is largely attributed to its substantial impact on patient outcomes. Once *K. pneumoniae *and other Gram-negative bacteria acquire resistance to colistin, the range of effective treatment options becomes considerably narrowed, frequently leaving healthcare professionals with limited antimicrobial choices. This reduction in therapeutic availability may lead to delayed administration of effective treatment, increased likelihood of treatment failure, prolonged disease progression, and a greater probability of severe clinical complications. Multiple studies have reported that infections caused by colistin-resistant pathogens are associated with increased morbidity and mortality, particularly among critically ill patients, immunocompromised individuals, and patients with pre-existing comorbid conditions [11]. In many cases, clinicians may be required to administer combination antimicrobial regimens or alternative agents that are comparatively less effective and potentially more toxic, thereby complicating patient care and negatively affecting treatment outcomes.

The importance of colistin resistance becomes more evident in tertiary-care hospitals and advanced healthcare settings, where patients are frequently subjected to prolonged hospitalization, invasive procedures, and repeated antimicrobial exposure. Within these environments, severely ill patients are particularly susceptible to healthcare-associated infections caused by resistant microorganisms. The extensive and, in certain situations, inappropriate use of broad-spectrum antibiotics generates significant selective pressure, facilitating the emergence, survival, and dissemination of resistant bacterial strains, including colistin-resistant* K. pneumoniae*. As resistance rates continue to increase, healthcare professionals encounter greater difficulties in treating infections that were previously manageable, thereby placing additional burdens on hospital resources and infection prevention systems [7].

From the perspective of infection control, the increasing occurrence of colistin resistance presents substantial challenges to healthcare institutions. Resistant bacterial strains may rapidly spread through direct contact between patients, contaminated hospital environments, invasive medical devices, and insufficient infection prevention practices. Outbreaks involving colistin-resistant pathogens have been increasingly reported, especially in high-risk settings such as ICUs, where patients frequently undergo mechanical ventilation, urinary catheterization, central venous catheterization, and prolonged antimicrobial therapy. The persistence and circulation of resistant strains within healthcare environments further complicate infection control efforts and increase the risk of hospital-acquired transmission [12].

The public health importance of colistin resistance extends beyond individual patient care because it significantly contributes to the expanding global challenge of AMR. The emergence of colistin-resistant organisms reduces the effectiveness of currently available antimicrobial therapies and threatens progress achieved in the treatment of infectious diseases. Since colistin often serves as one of the last available treatment options for infections caused by CRE, the development of resistance raises serious concerns regarding the emergence of pan-drug-resistant pathogens for which no effective treatment may remain available. Consequently, healthcare authorities and international organizations have expressed growing concern regarding the potential for widespread dissemination of resistant organisms across hospitals and communities [7].

Certain patient populations remain at particularly elevated risk of acquiring infections caused by colistin-resistant organisms. Patients admitted to ICUs, individuals undergoing major surgical interventions, those requiring invasive medical procedures, and patients receiving prolonged or repeated antibiotic treatment are particularly vulnerable to resistant infections [2]. Critically ill patients are commonly exposed to prolonged hospitalization, extensive use of broad-spectrum antibiotics, and immunosuppressive conditions, all of which contribute to increased susceptibility to bacterial colonization and infection. Additionally, the presence of indwelling medical devices, including urinary catheters, endotracheal tubes, and central venous catheters, further increases the risk of infection because these devices may act as potential sites for bacterial colonization and biofilm formation [2].

Apart from clinical complications, colistin-resistant organisms impose a substantial economic burden on healthcare systems. Resistant infections frequently require extended hospital stays, repeated microbiological investigations, sophisticated diagnostic procedures, and the administration of costly antimicrobial combinations. Moreover, prolonged ICU admissions, delayed recovery, and increased dependency on supportive medical care contribute considerably to healthcare expenditures, particularly in low- and middle-income countries [13]. Hospital outbreaks associated with resistant strains may further increase institutional costs because of the requirement for patient isolation, intensified surveillance, and enhanced infection control measures.

The increasing prevalence of colistin resistance also underscores the urgent need for effective ASPs aimed at promoting rational antibiotic use and minimizing unnecessary exposure to broad-spectrum antimicrobial agents. Appropriate antibiotic prescribing practices, stringent infection prevention measures, continuous microbiological monitoring, and early recognition of resistant isolates remain essential strategies for controlling the emergence and spread of resistance. In the absence of effective preventive interventions, the continued dissemination of colistin-resistant *K. pneumoniae* may further limit available therapeutic options and adversely affect patient outcomes in healthcare settings worldwide [12, 13]. Therefore, the significance of colistin resistance extends beyond its immediate effect on patient treatment and encompasses broader implications for healthcare systems, infection prevention strategies, antimicrobial stewardship, and global public health. A comprehensive understanding of the epidemiological and clinical importance of colistin resistance is essential for improving therapeutic decision-making, developing effective preventive strategies, and preserving the effectiveness of one of the final remaining antibiotics for the treatment of severe MDR bacterial infections [10, 13].

Emerging mechanisms of colistin resistance in* K. pneumoniae*

The emergence and widespread dissemination of colistin resistance in *K. pneumoniae* has become a major global healthcare challenge, mainly because of its growing association with MDR, extensively drug-resistant (XDR), and carbapenem-resistant strains. Colistin, a polymyxin-class antibiotic, has been reintroduced into clinical practice as a last-line therapeutic agent for infections caused by CRE, particularly carbapenem-resistant *K. pneumoniae* (CRKP). Nevertheless, the increasing administration of colistin in ICUs and tertiary-care hospitals among immunocompromised patients has accelerated the evolution of resistance mechanisms, thereby reducing the long-term effectiveness of this crucial antimicrobial drug [13].

Colistin resistance in *K. pneumoniae* develops through multiple molecular, biochemical, physiological, and adaptive mechanisms that impair the antibiotic's ability to interact with and damage the bacterial outer membrane. The bactericidal effect of colistin mainly depends on its interaction with the LPS layer of Gram-negative bacteria, especially the lipid A region, which acts as the primary target site. Under normal physiological circumstances, the positively charged cyclic peptide structure of colistin binds electrostatically to negatively charged phosphate groups present on lipid A. This interaction causes destabilization of the bacterial membrane by replacing divalent cations such as magnesium (Mg²⁺) and calcium (Ca²⁺), ultimately resulting in membrane disruption, leakage of intracellular components, and bacterial cell death.

Among the best-characterized and clinically important resistance mechanisms are structural modifications occurring in the lipid A portion of the LPS molecule. Resistant strains modify the bacterial surface charge through the incorporation of positively charged compounds such as phosphoethanolamine (pEtN) and 4-amino-4-deoxy-L-arabinose (L-Ara4N) into lipid A. These alterations reduce the negative charge of the outer membrane and significantly weaken the electrostatic interaction between colistin and lipid A, thereby lowering the drug's binding capacity and antimicrobial activity [14]. As a result, colistin is unable to effectively penetrate the bacterial membrane, enabling survival even in the presence of therapeutic concentrations of the antibiotic.

Lipid A modifications are predominantly regulated through chromosomal two-component regulatory systems (TCS), including PhoP/PhoQ, PmrA/PmrB, and CrrA/CrrB, which respond to environmental stressors such as magnesium deficiency, acidic conditions, oxidative stress, and antimicrobial exposure. These regulatory pathways control the expression of genes involved in membrane remodeling and resistance development. Among these mechanisms, mutations, insertional disruptions, or inactivation of the mgrB gene are considered one of the most frequent causes of colistin resistance in clinical isolates of *K. pneumoniae*. Normally, the mgrB gene acts as a negative feedback regulator of the PhoP/PhoQ signaling system. However, when this gene becomes altered or non-functional, persistent activation of the PhoP/PhoQ pathway occurs, leading to enhanced lipid A modification and increased resistance to colistin. Several reports have shown that insertion sequence-mediated disruption of mgrB is highly prevalent among colistin-resistant isolates obtained from ICUs and tertiary-care healthcare facilities [14].

Over recent years, plasmid-mediated resistance has emerged as a particularly concerning contributor to the spread of colistin resistance. The identification of *mcr* genes has substantially changed the epidemiological landscape of AMR because of their ability to spread between bacterial species through horizontal gene transfer. Members of the *mcr* family, including mcr-1, mcr-2, mcr-3, mcr-4, and mcr-5, encode pEtN transferase enzymes that promote the addition of pEtN to lipid A. Similar to chromosomal mechanisms, this process reduces the net negative charge of the bacterial membrane, thereby decreasing susceptibility to colistin. However, unlike chromosomal mutations, plasmid-borne *mcr* genes are highly transferable and can disseminate rapidly through bacterial conjugation, particularly in healthcare environments exposed to high antimicrobial pressure. The spread of *mcr* genes among enterobacterales represents a significant public health threat because it enables resistance transmission to previously susceptible bacterial populations, increasing the likelihood of outbreaks caused by pan-drug-resistant organisms [15].

Apart from lipid A modification, additional auxiliary and adaptive mechanisms also contribute to reduced susceptibility to colistin in *K. pneumoniae*. One such mechanism involves overproduction of capsular polysaccharides (CPS), which forms a thick extracellular layer surrounding bacterial cells. This hypercapsular structure acts as a protective barrier that limits antibiotic penetration and reduces direct contact between colistin molecules and the bacterial membrane. Consequently, hypervirulent strains of *K. pneumoniae*, which are frequently associated with increased capsule production, may exhibit greater tolerance to polymyxin antibiotics [16].

The contribution of efflux pumps to colistin resistance has also attracted increasing scientific interest. These systems function by actively expelling antimicrobial agents from bacterial cells, thereby decreasing intracellular drug concentrations to subtherapeutic levels. Although efflux-mediated resistance is generally regarded as less important than LPS modification, overexpression of multidrug efflux systems may work synergistically with chromosomal mutations to promote reduced susceptibility and the development of high-level resistance [16].

Alterations affecting outer membrane proteins, also known as porins, constitute another contributing resistance mechanism. Porins facilitate the movement of nutrients and antimicrobial agents across the bacterial membrane. Structural changes, altered expression, or complete loss of porins can reduce membrane permeability, thereby decreasing intracellular accumulation of colistin and other antibiotics. In combination with lipid A modifications, these porin alterations may considerably improve bacterial survival under antibiotic stress [16].

Biofilm formation has also emerged as an important mechanism associated with colistin resistance in *K. pneumoniae*. Biofilms are organized microbial communities embedded within a self-produced extracellular polymeric matrix that adheres to biological and non-biological surfaces, including urinary catheters, ventilators, endotracheal tubes, and central venous catheters. Bacteria residing within biofilms demonstrate markedly lower susceptibility to antimicrobial agents due to limited drug penetration, metabolic alterations, nutrient restriction, and the persistence of dormant bacterial cells. Biofilm-related infections are particularly problematic in healthcare environments because they contribute to chronic infection, prolonged hospital stay, and therapeutic failure. Several studies indicate that biofilm-producing *K. pneumoniae* isolates display decreased susceptibility to colistin and other last-resort antibiotics, thereby complicating clinical management [16, 17].

Recent studies have further highlighted the contribution of hetero-resistance and adaptive resistance in the progression of colistin resistance. Hetero resistance describes the presence of both susceptible and resistant bacterial subpopulations within the same isolate, which may contribute to treatment failure despite laboratory findings suggesting susceptibility. In contrast, adaptive resistance involves reversible physiological responses triggered by environmental stress or temporary antibiotic exposure, allowing bacterial survival in the absence of permanent genetic changes. These emerging resistance mechanisms present important diagnostic concerns because conventional susceptibility testing may fail to identify low-frequency resistant subpopulations [16, 17].

The presence of multiple resistance pathways in *K. pneumoniae* is particularly alarming in tertiary-care hospitals and ICUs, where the extensive use of broad-spectrum antibiotics creates strong selective pressure favoring resistant strains. Clinical factors such as prolonged hospitalization, invasive medical interventions, mechanical ventilation, immunosuppression, and inappropriate antibiotic use significantly contribute to the persistence and spread of resistant isolates within healthcare institutions. Furthermore, the coexistence of carbapenem and colistin resistance has substantially restricted available therapeutic options, frequently leaving clinicians with limited and potentially toxic alternatives [13]. Therefore, an in-depth understanding of both established and emerging mechanisms of colistin resistance is essential for accurate laboratory identification, molecular surveillance, infection prevention, rational antimicrobial therapy, and the implementation of effective antimicrobial stewardship strategies. Early detection of resistance determinants, together with continuous genomic monitoring, is crucial to limiting resistance dissemination and preserving the therapeutic value of colistin as one of the few remaining treatment options for severe infections caused by MDR* K. pneumoniae* [13-17].

Molecular basis and genetic determinants of colistin resistance

Beyond chromosomal mutations, contemporary research has elucidated several distinct genetic pathways driving the development and spread of colistin resistance in* K. pneumoniae* [5, 12, 18]. Disruption of the mgrB gene represents one of the primary mechanisms identified in clinical strains [3, 8]. Because mgrB functions as a negative regulator of the Phop/PhoQ signaling cascade, its mutation or functional inactivation triggers downstream lipid A alterations, which subsequently diminish colistin's binding affinity [9, 12]. Simultaneously, plasmid-borne *mcr* gene--spanning variants mcr-1 through mcr-10--have emerged as a critical concern due to their capacity for rapid interspecies dissemination via horizontal gene transfer [8, 9, 12]. The spread of these plasmid-mediated factors is especially problematic in clinical settings, where mobile genetic elements accelerate the transmission of resistance traits across diverse bacterial populations [12]. Furthermore, recent literature underscores how biofilm architecture restricts antibiotic penetration and promotes bacterial resilience when exposed to antimicrobial pressure [16, 17]. Ultimately, mapping these precise molecular mechanisms is crucial for refining diagnostic protocols and designing targeted therapeutic regimens [5, 12].

Phenotypic patterns of colistin resistance

The phenotypic pattern associated with colistin resistance in* K. pneumoniae *is typically determined using testing for antimicrobial susceptibility methods that assess the organism's growth response when exposed to the antibiotic. Clinically, isolates are categorized as susceptible, intermediate, or resistant based on the minimum inhibitory concentration (MIC) value measurements established by standard guidelines such as the Clinical and Laboratory Standards Institute (CLSI). Phenotypically resistant strains in *K. pneumoniae* commonly exhibit elevated MIC values, usually ≥4 μg/mL, indicating decreased sensitivity to colistin [6]. These resistant isolates are frequently classified as MDR and XDR or pandrug-resistant (PDR). These resistant isolates often show resistance not only to colistin but also to different classes of antibiotics, such as carbapenems, cephalosporins, fluoroquinolones, and aminoglycosides [9]. In laboratory settings, phenotypic resistance may be detected using methods such as broth microdilution. *K. pneumoniae* virulence factors include a polysaccharide capsule, biofilm formation, siderophore-mediated iron acquisition systems, adhesins, and LPS, which together enhance immune evasion, host colonization, and persistence, while antimicrobial susceptibility testing is commonly performed using the broth microdilution method to determine MICs, agar dilution, automated susceptibility testing systems, and rapid screening tests [9, 19]. Some isolates may display heteroresistance, a phenomenon in which a subpopulation of bacterial cells exhibits higher resistance levels than the main population, leading to treatment failure and diagnostic challenges [11]. The development of these phenotypic resistance patterns among* K. pneumoniae *isolates obtained from tertiary-care hospitals is a significant clinical concern, as it limits therapeutic options, prolongs duration of hospitalization, raises healthcare expenses, and contributes to higher morbidity and mortality among hospitalized patients [2].

Risk factors for colistin resistance in *K. pneumoniae*

The occurrence of colistin-resistant *K. pneumoniae *in tertiary-care hospitals is closely linked to a variety of clinical, therapeutic, and healthcare-associated risk factors that contribute to bacterial colonization, persistence, and infection. Understanding these contributing factors is essential for reducing the spread of resistance, improving clinical outcomes, and implementing effective infection prevention strategies. Since tertiary-care healthcare institutions commonly treat critically ill patients requiring prolonged hospitalization and extensive antimicrobial therapy, these settings provide suitable conditions for the emergence and spread of colistin-resistant organisms [2].

Prolonged hospitalization is considered one of the most important risk factors associated with colistin-resistant *K. pneumoniae.* Patients who experience extended hospital stays are more likely to be repeatedly exposed to healthcare-associated pathogens, contaminated hospital surroundings, invasive procedures, and prolonged antimicrobial treatment. Extended duration of hospitalization also increases the chances of bacterial colonization, particularly in hospital wards where MDR organisms are frequently encountered. Continuous interaction with healthcare personnel, prolonged dependence on medical devices, and repeated exposure to contaminated surfaces may additionally contribute to the acquisition and transmission of resistant pathogens [2]. In tertiary-care settings, prolonged hospital admission often reflects severe disease and repeated medical interventions, thereby increasing susceptibility to resistant infections.

Admission to ICUs represents another significant risk factor for acquiring colistin-resistant* K. pneumoniae.* Critically ill patients admitted to ICUs are particularly vulnerable because they frequently require advanced supportive interventions such as prolonged mechanical ventilation, urinary catheterization, central venous catheterization, and invasive monitoring procedures. Furthermore, ICU patients often receive prolonged treatment with broad-spectrum antimicrobial agents, which creates considerable selective pressure that favors the persistence and proliferation of resistant bacterial populations. ICU environments also increase opportunities for cross-transmission due to close patient contact, frequent interaction with healthcare workers, and the use of shared medical devices [2]. As a result, ICUs are widely recognized as major reservoirs for healthcare-associated MDR infections.

Previous exposure to broad-spectrum antimicrobial agents is regarded as one of the strongest predictors for the emergence of colistin-resistant *K. pneumoniae*. The extensive use of antibiotics, especially broad-spectrum agents, disrupts normal microbial flora and generates selective pressure that supports the survival and multiplication of resistant bacteria. Repeated or prolonged antibiotic administration may eliminate susceptible microorganisms while promoting the dominance of resistant strains within hospital settings. Consequently, patients receiving multiple courses of antimicrobial therapy are more susceptible to infections caused by organisms that possess resistance mechanisms [2]. Inappropriate empirical treatment and excessive antibiotic use may further intensify the selection of resistant organisms, contributing to the increasing prevalence of resistance in healthcare environments.

The use of invasive medical devices, such as urinary catheters, mechanical ventilators, central venous catheters, and endotracheal tubes, also serves as a major risk factor for colistin-resistant infections. These medical devices can compromise natural host defense mechanisms and create direct entry routes for microorganisms into sterile anatomical sites. Additionally, indwelling devices often provide surfaces for bacterial adherence and biofilm development, allowing microorganisms to persist despite exposure to antimicrobial therapy. Mechanical ventilation is strongly associated with ventilator-associated pneumonia, whereas urinary catheterization increases the risk of catheter-associated UTIs caused by resistant pathogens [2]. Prolonged placement of such invasive devices further increases the likelihood of bacterial colonization and persistent infection.

Patients with severe underlying medical conditions and weakened immune systems are at greater risk of developing infections caused by colistin-resistant* K. pneumoniae*. Individuals affected by chronic illnesses, including diabetes mellitus, malignancies, chronic kidney disease, liver disease, and cardiovascular disorders, often exhibit impaired immune function and frequent healthcare exposure, increasing their susceptibility to resistant infections. Likewise, immunocompromised patients, including organ transplant recipients, cancer patients receiving chemotherapy, and individuals undergoing corticosteroid or immunosuppressive therapy, are particularly susceptible because of weakened host defense mechanisms. These patient populations commonly require prolonged hospitalization and repeated antimicrobial exposure, both of which increase the risk of acquiring resistant organisms [2].

A prior history of hospitalization also significantly increases the likelihood of infection with colistin-resistant strains. Patients with repeated hospital admissions are more likely to have previous exposure to resistant microorganisms, prolonged antibiotic treatment, and invasive medical interventions. Colonization with resistant pathogens may continue even after discharge from healthcare facilities, increasing the possibility of recolonization or recurrent infection during future hospital admissions. Hospitals with high rates of AMR may therefore act as major reservoirs for repeated exposure to resistant organisms [2].

Frequent or inappropriate use of last-line antimicrobial agents, particularly colistin and carbapenems, is recognized as an important contributor to the emergence and selection of resistant *K. pneumoniae* strains. Overdependence on these antibiotics, especially during empirical treatment or prolonged therapy, creates strong selective pressure that encourages the survival of bacteria capable of developing resistance mechanisms. Improper dosing schedules, incomplete treatment courses, and unnecessary administration of antimicrobial agents may further contribute to the selection and persistence of resistant bacterial populations [13]. Since colistin is frequently used as a therapeutic option for severe infections caused by carbapenem-resistant pathogens, increased dependence on this antibiotic has accelerated resistance development in tertiary-care healthcare facilities.

In cases involving CRKP, biofilm formation is considered another clinically important risk factor contributing to AMR. The ability of *K. pneumoniae* to produce biofilms allows bacterial cells to attach to biological tissues and medical devices while remaining protected within a self-produced extracellular polymeric matrix. Bacteria embedded within biofilms demonstrate significantly decreased susceptibility to antimicrobial agents, including colistin, due to limited drug penetration, altered metabolic activity, and the presence of dormant persister cells. Biofilms commonly develop on urinary catheters, ventilators, endotracheal tubes, and vascular devices, thereby promoting persistent infection and reducing treatment efficacy [17].

The transmission of resistant organisms within healthcare settings also contributes substantially to the spread of colistin-resistant *K. pneumoniae*. Inadequate infection prevention practices, poor hand hygiene compliance, contaminated hospital environments, colonized healthcare personnel, and insufficient sterilization of medical equipment may facilitate transmission of resistant strains between patients. Outbreaks involving resistant* K. pneumoniae* are particularly concerning in tertiary-care healthcare institutions because of the large number of vulnerable patients requiring specialized medical care [17]. The simultaneous presence of multiple risk factors in these settings considerably increases the possibility of widespread dissemination and persistent bacterial colonization.

Therefore, recognizing and continuously monitoring the risk factors associated with colistin-resistant *K. pneumoniae* is essential for implementing effective infection prevention and control strategies in tertiary-care hospitals. Strengthening antimicrobial stewardship initiatives, encouraging rational antibiotic use, reducing unnecessary dependence on invasive medical devices, maintaining strict hand hygiene practices, and performing regular microbiological surveillance are important approaches for reducing the burden of resistant infections. Early identification of high-risk patients may further help healthcare professionals implement targeted preventive measures aimed at limiting the emergence and spread of resistant organisms within healthcare environments [2, 13, 17].

Clinical importance of colistin resistance in *K. pneumoniae*

The rising occurrence of colistin-resistant *K. pneumoniae* has become a significant clinical problem in tertiary-care hospitals, especially in ICUs where MDR organisms are commonly encountered [13]. Colistin is frequently utilized as a last-line therapeutic agent for managing infections resulting from carbapenem-resistant Gram-negative bacteria [19]. The development of resistance to colistin greatly reduces the available treatment options and is linked to increased morbidity and mortality, longer durations of hospitalization, and elevated healthcare expenses. Infections due to resistant strains of* K. pneumoniae*, including bloodstream infections, pneumonia, and UTIs, commonly are linked to treatment failure and unfavorable clinical outcomes [2]. Moreover, the transmission of resistant strains within hospital environments presents a serious threat to patient safety and complicates infection control efforts [13]. Consequently, regular monitoring of AMR trends, adherence to strict infection prevention measures, and the effective execution of ASPs are necessary for minimizing the clinical effects of colistin-resistant infections in healthcare settings.

Therapeutic approaches for colistin-resistant *K. pneumoniae*

Managing infections triggered by colistin-resistant* K. pneumoniae* poses a formidable therapeutic dilemma due to the scarce availability of efficacious antimicrobial agents [9, 10]. For severe infections, clinicians increasingly favor combination antimicrobial regimens over monotherapy to optimize clinical outcomes and suppress further resistance development [10]. Multidrug regimens incorporating colistin, tigecycline, meropenem, aminoglycosides, or ceftazidime-avibactam have shown fluctuating clinical success, which is largely dictated by the specific susceptibility profile of the bacterial isolate [9, 10]. Nevertheless, therapeutic efficacy remains erratic, particularly in critically ill populations and individuals suffering from bloodstream infections [9]. The escalating prevalence of XDR and PDR strains further obfuscates clinical management, highlighting the pressing requirement for the discovery of novel antimicrobial agents and innovative therapeutic strategies [6, 8].

Infection prevention and antimicrobial stewardship

Effective infection prevention and antimicrobial stewardship strategies play a fundamental role in reducing the transmission and limiting the emergence of colistin-resistant *K. pneumoniae* within healthcare settings, particularly in tertiary-care hospitals and ICUs, where the burden of MDR organisms remains considerably high [2, 13]. Since colistin-resistant strains are frequently associated with healthcare-associated infections and prolonged hospital stays, the implementation of comprehensive infection prevention measures combined with rational antimicrobial use is essential for controlling the spread of resistance and improving patient outcomes. The growing prevalence of resistant organisms has emphasized the need for coordinated institutional strategies that focus on early detection, prevention of transmission, and optimization of antimicrobial therapy [2, 13].

Infection prevention measures are critical for interrupting the transmission of colistin-resistant *K. pneumoniae* within healthcare environments. Among these strategies, strict adherence to hand hygiene protocols remains one of the most effective and cost-efficient interventions for minimizing the spread of resistant pathogens. Proper hand hygiene practices by healthcare professionals, patients, and caregivers significantly reduce cross-contamination and limit direct patient-to-patient transmission. Compliance with recommended hand hygiene guidelines, including handwashing with soap and water or the use of alcohol-based hand sanitizers before and after patient contact, is particularly important in high-risk areas such as ICUs, surgical wards, and transplant units, where vulnerable patient populations are concentrated [4,13].

Environmental cleaning and hospital disinfection procedures also represent essential components of infection prevention programs. Resistant *K. pneumoniae* strains may survive on contaminated hospital surfaces, medical equipment, and frequently touched objects for prolonged periods, thereby increasing the likelihood of indirect transmission. Routine environmental disinfection of patient care areas, ICUs, ventilators, bed rails, medical instruments, and frequently contacted surfaces is necessary to reduce environmental reservoirs of resistant organisms. The use of standardized cleaning protocols and appropriate disinfectants contributes significantly to minimizing healthcare-associated transmission and reducing hospital-acquired infections caused by resistant strains [4, 13].

Contact isolation precautions are equally important for preventing the spread of colistin-resistant* K. pneumoniae*, particularly among hospitalized patients with confirmed or suspected colonization or infection. Isolation strategies commonly include placement of infected patients in separate rooms or designated cohort areas, the use of personal protective equipment (PPE) such as gloves and gowns by healthcare workers, and restriction of unnecessary patient movement within healthcare facilities. These measures reduce the risk of cross-transmission to susceptible individuals and help prevent hospital outbreaks involving resistant pathogens. In tertiary-care hospitals and ICUs, where critically ill patients are particularly vulnerable, strict implementation of contact precautions remains essential for infection containment [13, 19].

Active surveillance and screening programs also play an important role in identifying patients colonized or infected with colistin-resistant organisms at an early stage. Screening high-risk patients, especially those admitted to ICUs, transferred from other healthcare facilities, or with a prior history of prolonged antibiotic use, can facilitate timely detection and isolation of resistant strains. Microbiological surveillance through routine culture testing and antimicrobial susceptibility analysis allows healthcare institutions to monitor trends in resistance patterns and identify potential outbreaks before widespread dissemination occurs. Early recognition of resistant isolates is therefore crucial for implementing targeted infection prevention measures and minimizing healthcare-associated transmission [13, 19].

In addition to infection prevention measures, ASPs are recognized as one of the most effective strategies for controlling AMR and preventing the emergence of colistin-resistant *K. pneumoniae.* These programs aim to optimize antimicrobial prescribing practices, reduce unnecessary antibiotic exposure, and ensure appropriate selection, dosing, and duration of antimicrobial therapy. Inappropriate and excessive administration of broad-spectrum antibiotics contributes substantially to selective pressure, encouraging the survival and spread of resistant bacterial populations. Consequently, antimicrobial stewardship interventions are essential for reducing resistance development and preserving the effectiveness of available therapeutic agents [20].

The rational and judicious use of last-resort antibiotics, including colistin and carbapenems, is particularly important within stewardship frameworks. Since these agents are frequently reserved for severe infections caused by MDR pathogens, unnecessary or inappropriate administration should be avoided to minimize selective pressure. Antimicrobial stewardship teams often emphasize evidence-based prescribing practices, culture-guided therapy, de-escalation strategies, and restriction policies for high-risk antibiotics to ensure their optimal use. Appropriate dose optimization and therapeutic monitoring may further improve treatment outcomes while reducing the likelihood of resistance emergence [2, 13].

Educational initiatives and multidisciplinary collaboration are additional important aspects of infection prevention and antimicrobial stewardship. Continuous training of healthcare professionals regarding infection control protocols, rational antibiotic use, and emerging resistance patterns may improve adherence to preventive practices and prescribing guidelines. Collaboration among microbiologists, infectious disease specialists, pharmacists, intensivists, nurses, and infection control teams is necessary to establish coordinated interventions aimed at reducing the transmission of resistant organisms and improving antimicrobial use [20].

In high-risk healthcare settings such as ICUs, the integration of infection prevention strategies with antimicrobial stewardship efforts is particularly important because critically ill patients frequently require prolonged hospitalization, invasive medical procedures, and repeated antibiotic exposure. These conditions create an environment favorable for the emergence and dissemination of resistant strains. Therefore, combining rigorous infection control measures with optimized antimicrobial prescribing may significantly reduce healthcare-associated transmission and prevent hospital outbreaks involving colistin-resistant* K. pneumoniae *[4, 13].

Therefore, effective infection prevention and antimicrobial stewardship remain indispensable for controlling the spread of colistin-resistant* K. pneumoniae *in healthcare environments. Strict compliance with hand hygiene, environmental disinfection, contact isolation precautions, active screening, and microbiological surveillance, together with rational antimicrobial prescribing and stewardship interventions, are essential strategies for minimizing resistance development and preventing the dissemination of resistant strains. Sustained institutional commitment toward infection prevention and antimicrobial stewardship is crucial for preserving the effectiveness of last-resort antibiotics and reducing the growing burden of AMR in tertiary-care healthcare settings [3, 4, 13, 20, 21].

## Conclusions

In conclusion, the characterization of the phenotypic patterns of colistin resistance in* K. pneumoniae* isolates in a tertiary-care hospital highlights the growing concern in healthcare settings. The development of resistance to last-resort antibiotics such as colistin significantly restricts treatment options for infections caused by MDR and carbapenem-resistant organisms. The occurrence of resistant strains is commonly linked with extended hospitalization, admission to the ICU, and prior exposure to broad-spectrum antibiotics. Continuous monitoring of antimicrobial susceptibility patterns and early detection of resistant isolates are essential for effective patient management and prevention of treatment failure. Implementation of strict infection prevention measures, appropriate antibiotic use, and ASPs is essential to minimize the spread of resistant *K. pneumoniae* strains in hospital environments. Furthermore, ongoing surveillance and research are essential for understanding resistance trends and for facilitating the progression of new therapeutic strategies to address AMR within tertiary-care healthcare facilities.
